# Septins: Regulators of Protein Stability

**DOI:** 10.3389/fcell.2016.00143

**Published:** 2016-12-20

**Authors:** Olga Vagin, David O. Beenhouwer

**Affiliations:** ^1^Department of Physiology, Geffen School of Medicine at UCLALos Angeles, CA, USA; ^2^VA Greater Los Angeles Healthcare SystemLos Angeles, CA, USA; ^3^Department of Medicine, Geffen School of Medicine at UCLALos Angeles, CA, USA; ^4^Division of Infectious Diseases, VA Greater Los Angeles Health Care SystemLos Angeles, CA, USA

**Keywords:** septins, protein stability, botulinum toxins, receptor tyrosine kinases, hypoxia-inducible factor 1α

## Abstract

Septins are small GTPases that play a role in several important cellular processes. In this review, we focus on the roles of septins in protein stabilization. Septins may regulate protein stability by: (1) interacting with proteins involved in degradation pathways, (2) regulating the interaction between transmembrane proteins and cytoskeletal proteins, (3) affecting the mobility of transmembrane proteins in lipid bilayers, and (4) modulating the interaction of proteins with their adaptor or signaling proteins. In this context, we discuss the role of septins in protecting four different proteins from degradation. First we consider botulinum neurotoxin serotype A (BoNT/A) and the contribution of septins to its extraordinarily long intracellular persistence. Next, we discuss the role of septins in stabilizing the receptor tyrosine kinases EGFR and ErbB2. Finally, we consider the contribution of septins in protecting hypoxia-inducible factor 1α (HIF-1α) from degradation.

## Introduction

Protein turnover is regulated by intracellular degradation pathways, including the ubiquitin-proteasome and lysosome-autophagy systems. Protein ubiquitylation plays a key role in the regulation of protein degradation. It is well-established that polyubiquitin chains, predominantly but not exclusively with Lys48 linkages, target proteins to proteasomal degradation (Peng et al., 2003; Xu et al., 2009; Kaiser et al., 2011; Kim et al., 2011). Ubiquitylation also acts as a signal to endocytosis (Terrell et al., 1998; Mukhopadhyay and Riezman, 2007) or protein sorting of internalized proteins to multi-vesicular bodies followed by lysosomal degradation (Raiborg and Stenmark, 2009; Ren and Hurley, 2010; Stringer and Piper, 2011). In addition, ubiquitylation regulates signaling by modulating protein-protein interactions (Mukhopadhyay and Riezman, 2007; Yau and Rape, 2016), which may affect localization of the protein and hence its susceptibility to degradation.

The half-life of most cellular proteins is typically a few days (~40–69 h) (Varshavsky, 1996). Soluble proteins usually have a relatively short half-life (several hours), while transmembrane proteins tend to survive longer (2–3 days). At the extremes, some proteins, like hypoxia-inducible factor 1α (HIF-1α), last only seconds (Yu et al., 1998), while others, like the light chain of serotype A botulinum neurotoxin, remain intact for several months (Dolly and Aoki, 2006).

Septins are small GTPases that form hetero-oligomeric structures and act as linkers between the plasma membrane and the intracellular cytoskeleton. Through interactions with transmembrane and cytosolic proteins, septins organize segregated membrane microdomains, and membrane-associated protein complexes. The contribution of septins to the regulation of numerous cellular processes, including cell division, protein trafficking, exocytosis, cell migration, and cell proliferation, has been described in several excellent reviews (Hall and Russell, 2012; Mostowy and Cossart, 2012; Dolat et al., 2014; Fung et al., 2014). In this review, we will focus on the roles of septins in the regulation of protein stability.

Septins regulate protein stability by affecting several intracellular processes. First, they interact with components of endocytosis and exocytosis machinery (Beites et al., 1999, 2005; Amin et al., 2008; Maimaitiyiming et al., 2013; Phan et al., 2013; Tokhtaeva et al., 2015; Song et al., 2016). Second, they may regulate the interaction between transmembrane proteins and cytoskeletal proteins (Gilden and Krummel, 2010; Hagiwara et al., 2011; Hall and Russell, 2012; Mostowy and Cossart, 2012; Bridges et al., 2016). Third, by forming diffusion barriers, septins can modulate the mobility of transmembrane proteins in lipid bilayers (Hagiwara et al., 2011; Saarikangas and Barral, 2011; Hall and Russell, 2012; Mostowy and Cossart, 2012; Fung et al., 2014; Bridges et al., 2016). Fourth, septins form scaffolds and thus can modulate the interaction of proteins with their adaptor or signaling proteins (Ihara et al., 2007; Spiliotis and Gladfelter, 2011; Ageta-Ishihara et al., 2013; Ghossoub et al., 2013). All these processes can affect trafficking and sorting of proteins to degradative pathways. Finally, septins interact with proteins involved in degradation pathways, including ubiquitin ligases, and de-ubiquitylating enzymes, thus modulating protein turnover rate (Nakahira et al., 2010; Diesenberg et al., 2015; Marcus et al., 2016).

## Septins contribute to the remarkable stability of botulinum neurotoxin a light chain

### Botulinum neurotoxins and their stability

Botulism is a life-threatening illness caused by neurotoxins produced by *Clostridium botulinum*. Botulinum neurotoxins (BoNTs) are synthesized as a single-chain 150 kDa polypeptide that is later cleaved by proteases for biological activity into heavy (100 kDa) and light (50 kDa) chains. BoNTs bind through the heavy chain to specific receptors on motor neurons followed by receptor-mediated endocytosis and translocation of their light chains into the cytoplasm (Montal, 2010). Once inside the neuron, the light chain acts as a zinc-dependent endoprotease to cleave one of the SNARE (Soluble *N*-ethylmaleimide-sensitive factor Attachment Protein REceptor) proteins involved in vesicle-membrane fusion. This prevents release of acetylcholine into the synaptic cleft, resulting in neuromuscular paralysis (Dolly and Aoki, 2006; Popoff and Bouvet, 2009; Montal, 2010). As a result, patients with botulism develop severe muscle weakness. The illness may progress to total loss of muscle function, inability to breathe, and death unless supportive care is provided (Arnon et al., 2001).

Of the seven serotypes of BoNT (A–G), human botulism is caused by serotypes A, B, E and rarely by F (Arnon et al., 2001). BoNT/A intoxication lasts surprisingly long: even after 57 weeks following exposure to BoNT/A, humans may still demonstrate 22% muscle paralysis (Eleopra et al., 1998). Current treatment for adult botulism consists of supportive care and passive immunization with heptavalent equine antitoxin (Centers for Disease and Prevention, 2010). Antitoxin lowers death rates and shortens duration of symptoms only if administered within 24 h of disease onset (Arnon et al., 2001; Sobel, 2005). However, antitoxin does not enter neurons and does not reverse paralysis; hence, there is no specific treatment that targets BoNT once it is inside motor neurons.

While they cleave the same SNARE protein (SNAP-25), BoNT/A causes much longer duration of paralysis than BoNT/E (Keller et al., 1999; Adler et al., 2001; Bajohrs et al., 2004). In cultured spinal cord neurons, the proteolytic activity of BoNT/A, and BoNT/E persists for more than 80 days and <1 day, respectively (Keller et al., 1999). When expressed in cultured neuronal cells, the light chain of BoNT/A (LCA) survives significantly longer than the light chain of BoNT/E (LCE) due to less efficient degradation (Fernández-Salas et al., 2004; Tsai et al., 2010; Vagin et al., 2014). However, LCA is cleaved by proteases *in vitro* as efficiently as LCE (Gimenez and DasGupta, 1993; Beecher and DasGupta, 1997; Prabakaran et al., 2001). Thus, intrinsic resistance to intracellular proteases does not explain LCA persistence.

### Septins protect botulinum toxin light chain a from intracellular degradation

Even though LCA does not possess a transmembrane domain, it is localized in clusters almost exclusively at the plasma membrane (Vagin et al., 2014). In contrast, LCE remains in the cytoplasm, where its half-life corresponds to that of typical cytoplasmic proteins (Foran et al., 2003; Fernández-Salas et al., 2004). A double mutation, L428A/L429A, in a dileucine-containing motif (EFYKLL), which is present in LCA but not in LCE, decreases association of LCA with the plasma membrane (Fernández-Salas et al., 2004), prevents its clustered distribution, and shortens the half-life of LCA in cultured neuronal cells (Vagin et al., 2014) and shortens neuroparalytic effects of BoNT/A in mice (Wang et al., 2011). Furthermore, fusion of LCE with BoNT/A stabilizes LCE, while L428A/L429A mutation in the LCA portion of this chimera reverts LCE back into a short-lived protein (Wang et al., 2011). Mass spectrometry analysis identified septins as proteins that interact with LCA but not with the L428A/L429A LCA mutant (Table 1) (Vagin et al., 2014). Septins co-localize with LCA in plasma membrane-associated clusters, and the L428A/L429A mutation decreases this co-localization and accelerates ubiquitylation-dependent degradation of LCA (Vagin et al., 2014). Similarly, impairment of septin oligomerization with forchlorfenuron (FCF), decreases LCA clustering, and increases LCA degradation (Vagin et al., 2014). Therefore, the dileucine-mediated formation of membrane-attached LCA- and septin-containing complexes is crucial for the long-lasting stabilization of LCA and LCA-related neuroparalytic activity. The involvement of septins in LCA stabilization is consistent with the reports on enrichment of septins in the presynaptic sites in neurons (Kinoshita et al., 2000; Xue et al., 2004; Yang et al., 2010; Tsang et al., 2011) and interaction of septins with SNARE proteins (Beites et al., 1999; Dent et al., 2002; Ihara et al., 2007; Ito et al., 2009; Wasik et al., 2012; Tokhtaeva et al., 2015).

**Table 1:** **Septin-mediated regulation of protein stability**.

**Septin- regulated protein**	**Septin(s) involved in regulation**	**Protein-septin interaction**	**Effect(s) of septins on protein stability**	**Molecular mechanism of septin-mediated effect**	**References**
LCA	Septin 2 Septin 3 Septin 5 Septin 6 Septin 7 Septin 9 Septin 11	Dileucine motif (428L/429L) required for binding	Protect from ubiquitylation-dependent degradation	Unknown	Vagin et al., 2014
EGFR	Septin 9 Septin 2 Septin 7	Not demonstrated	Protects from ubiquitylation and degradation	Septin 9 competes with CBL for binding to CIN85	Diesenberg et al., 2015
ErbB2	Septin 2 Septin 9 Septin 7	Multiprotein complex with several septins	Protect from ubiquitylation and lysosomal degradation	Unknown	Marcus et al., 2016
HIF-1α	Septin 9	GTPase domain of septin 9 required for interaction	Protects from ubiquitylation and degradation	Septin 9 competes for RACK1 binding to HIF-1α	Amir et al., 2006, 2009; Golan and Mabjeesh, 2013; Vardi-Oknin et al., 2013
MET	Septin 2 Septin 11	Unknown	Differently modulate surface expression and association with the cytoskeleton	Unknown	Mostowy et al., 2011
JNK	Septin 9	GTPase domain of septin 9 required for interaction	Protects from degradation	Unknown	Gonzalez et al., 2009

## Septins stabilize receptor tyrosine kinases

### Persistent signaling of receptor tyrosine kinases

EGFR family receptor tyrosine kinases (ErbB1 or EGFR, ErbB2, ErbB3, ErbB4) trigger signaling cascades that regulate critical cellular processes, such as growth, differentiation, proliferation, adhesion, survival, and migration (Blume-Jensen and Hunter, 2001). One of the primary mechanisms that regulates the duration of downstream signaling after activation is the removal of these receptors from the membrane by endocytosis followed by trafficking back to the cell surface or to lysosomes for degradation (Wiley and Burke, 2001; Sorkin and Goh, 2009). Over-expression and aberrant degradation of ErbB receptors lead to enhanced or continuous signaling, promoting malignant transformation (Sangwan and Park, 2006; Lemmon and Schlessinger, 2010; Tebbutt et al., 2013; Arteaga and Engelman, 2014).

Endocytosis and degradative sorting of EGFR are regulated by the ubiquitin ligase CBL (Levkowitz et al., 1996; Schmidt and Dikic, 2005). EGFR stimulation by ligand binding triggers the recruitment of CBL, which induces multi-mono- and K63-polyubiquitylation of the receptor (Mosesson et al., 2003; Huang et al., 2006, 2013). Whether ubiquitylation is required for endocytosis is still a point of controversy. However, it is generally agreed that CBL-mediated receptor ubiquitylation targets internalized receptors for lysosomal degradation, while non-ubiquitylated receptors are recycled back to the membrane. CBL-interacting protein of 85 kDa (CIN85) mediates the interaction between CBL and EGFR and hence is involved in the downregulation of EGFR (Soubeyran et al., 2002; Haglund et al., 2003).

In contrast to EGFR, ErbB2 does not have a known ligand (Garrett et al., 2003) and is thought to be resistant to endocytic down-regulation (Wang et al., 1999; Haslekås et al., 2005; Roepstorff et al., 2008; Sorkin and Goh, 2008). Ubiquitylation is important for ErbB2 degradation (Vuong et al., 2013), but the involvement of CBL is not clear (Levkowitz et al., 1996; Klapper et al., 2000). Heat shock protein 90 (HSP90) is required for stable expression of ErbB2 at the plasma membrane, and disruption of ErbB2 association with HSP90 allows for recruitment of HSP70 and ubiquitin ligases CHIP and/or Cullin-5, leading to ErbB2 ubiquitylation (Xu et al., 2002; Ehrlich et al., 2009), internalization and lysosomal degradation (Tikhomirov and Carpenter, 2000; Austin et al., 2004; Lerdrup et al., 2006). Proteasomal activity is required for endocytosis and lysosomal targeting of ErbB2 but is not directly involved in ErbB2 proteolysis (Lerdrup et al., 2006, 2007; Roepstorff et al., 2008).

### Septins protect receptor tyrosine kinases from ubiquitylation, endocytosis and degradation

CIN85 is involved in down-regulation of EGFR by tethering CBL to the endocytic machinery in an EGF-dependent manner (Dikic, 2002). A number of studies have suggested that CIN85 participates in both the initial step of EGFR internalization (Soubeyran et al., 2002) and also in receptor trafficking and degradation (de Melker et al., 2001; Kowanetz et al., 2004; Schroeder et al., 2012). Knockdown of CIN85 results in a decrease in EGFR ubiquitylation (Rønning et al., 2011), while prevention of CIN85 phosphorylation affects efficient sorting and degradation of EGFR but has no effect on receptor endocytosis (Schroeder et al., 2012). A recent study has identified CIN85 as an interacting partner of septin 9 (Table 1) (Diesenberg et al., 2015). This interaction depends on the presence of a conserved ProArg motif in the N-terminus of septin 9 and mediates the formation of a multiprotein complex of CIN85 with septin 9 and other septins. *In vitro* binding studies suggest that septin 9 competes with CBL for binding to CIN85 (Diesenberg et al., 2015). Stimulation of EGFR with EGF in Hela cells results in the recruitment of the CIN85-septin complex to the plasma membrane. Depletion of septin 9 increases the degree of EGFR ubiquitylation and accelerates its degradation. Taken together, these data suggest that septin 9 negatively regulates EGFR degradation by preventing the association of the ubiquitin ligase CBL with CIN85, resulting in reduced EGFR ubiquitylation, and degradation (Diesenberg et al., 2015).

Another recent study has identified septins as interacting partners and regulators of the persistent expression of ErbB2 (Marcus et al., 2016). Several septins, including septin 2, septin 7, and septin 9, co-localize and interact with ErbB2 at the plasma membrane in gastric cancer cells. Inhibition of septin filament assembly-disassembly with FCF: (1) decreases association of septins with ErbB2, (2) reduces plasma-membrane localization of septins, (3) increases the amount of septin-free ErbB2, (4) induces ubiquitylation of ErbB2, and (5) accelerates its lysosomal degradation. A similar increase in ErbB2 degradation is observed in septin 2-depleted cells. These results imply that normally organized septin filaments protect ErbB2 from ubiquitylation, endocytosis, and lysosomal degradation.

This protective effect of septins is not related to the regulation of ErbB2 interaction with its chaperone HSP90 because the FCF-induced ubiquitylation and degradation of ErbB2 is not altered by geldanamycin (Marcus et al., 2016). This inhibitor down-regulates ErbB2 by disrupting the ErbB2-HSP90 interaction (Tikhomirov and Carpenter, 2000; Lerdrup et al., 2006, 2007). Therefore, distinct and complementary effects of FCF, and geldanamycin present a potential for augmented targeting of ErbB2 persistence in cancer.

FCF-induced ubiquitylation of ErbB2 is unlikely to be due to septin 9-mediated regulation of CBL as has been reported for EGFR (Diesenberg et al., 2015), because ErbB2 does not interact with CIN85 and FCF does not affect levels of EGFR (Marcus et al., 2016). The involvement of other ubiquitin ligases identified as septin interacting proteins (Nakahira et al., 2010; Tokhtaeva et al., 2015) in septin-regulated ErbB2 ubiquitylation has not been evaluated. Alternatively, septins may recruit de-ubiquitylating enzymes to ErbB2, resulting in a lower steady state level of ErbB2 ubiquitylation. In particular, ErbB2 interacts with a de-ubiquitylating enzyme USP9x (Marx et al., 2010; Marcus et al., 2016) that has been shown to protect ErbB2 from bortezomib-induced lysosomal degradation (Marx et al., 2010). In addition, septins play critical roles in endocytosis and exocytosis (Beites et al., 1999, 2005; Maimaitiyiming et al., 2013; Phan et al., 2013; Tokhtaeva et al., 2015; Song et al., 2016), and disruption of septin dynamics with FCF may induce ErbB2 internalization or delay its recycling to the plasma membrane.

## Septins stabilize hypoxia-inducible factor 1α

### Role of HIF-1α in cell responses to hypoxia

HIF-1 is a transcription factor and key regulator of cellular responses to changes in oxygen concentration that allow cell adaptation and survival under hypoxic conditions (Semenza, 2014). HIF-1 is composed of the oxygen-regulated subunit, HIF-1α, and the constitutively expressed HIF-1β subunit. HIF-1α is constantly produced and degraded under normoxic conditions due to oxygen-dependent hydroxylation, which promotes binding of von Hippel-Lindau protein (VHL), leading to ubiquitylation, and proteasomal degradation of HIF-1α. Under hypoxic conditions, HIF-1α is not hydroxylated, does not interact with VHL, translocates to the nucleus, and binds to hypoxia-response elements in target genes. The expression of over 70 genes is known to be activated at the transcriptional level by HIF-1 (Semenza, 2014). HIF-1α rapidly accumulates under hypoxic conditions and is degraded upon reoxygenation with a half-life of under 1 min (Yu et al., 1998). Another level of post-translational regulation does not depend on oxygen, hydroxylation, or VHL but requires the interaction between HIF-1α and RACK1 (receptor of activated protein C kinase). RACK1 competes with HSP90 for binding to HIF-1α and promotes the proteasome-dependent degradation of HIF-1α (Liu et al., 2007; Liu and Semenza, 2007). Activation of the HIF system has been observed in carcinogenesis and numerous cancers (Semenza, 2012, 2016a,b; Hubbi and Semenza, 2015). Increased levels of HIF-1 activity are often associated with increased tumor aggressiveness, therapeutic resistance, and mortality.

### Septin 9 protects HIF-1α from degradation

A search for HIF-1α-interacting proteins in human prostate cancer cells identified septin 9 (Sept9_v1 isoform) (Amir et al., 2006). Over-expression of septin 9 decreases ubiquitylation and degradation of HIF-1α and activates HIF-1α-dependent transcriptome (Amir et al., 2006). Co-immunoprecipitation experiments show that septin 9 competes with RACK1 for binding to HIF-1α (Amir et al., 2009). Inhibition of HSP90 induces RACK1-dependent HIF-1α degradation, and the rate of this degradation is significantly lower in cells over-expressing septin 9 (Amir et al., 2009). Taken together, these results implicate septin 9 in oxygen-independent stabilization of HIF-1α. As long as HIF-1α is bound to HSP90 or septin 9, it is protected from RACK1-mediated degradation via the proteasome. In the presence of HSP90 inhibitors, the HIF-1α-HSP90 association is disrupted, leading to a competition between RACK1, which promotes HIF-1α degradation, and septin 9, which confers HIF-1α stabilization (Amir et al., 2009). In support of this HIF-1α-stabilizing role of septin 9, disruption of normal assembly-disassembly of septin oligomers with FCF induces degradation of HIF-1α, and inhibition in HIF-1α transcriptional activity in various cancer cell types (Vardi-Oknin et al., 2013).

## Concluding remarks

Septins stabilize integral membrane proteins (EGFR and ErbB2), membrane-associated proteins (LCA), and cytosolic/nuclear proteins (HIF-1α) by attenuating their ubiquitylation and degradation. Septin 9 also interacts with and stabilizes another cytosolic/nuclear protein, c-Jun-N-terminal kinase (JNK) (Gonzalez et al., 2009), but whether septin 9 affects the degree of JNK ubiquitylation has not been investigated. Septin 2 and septin 11 regulate surface expression of MET receptor tyrosine kinase by modulating its mobility in the lipid bilayer and its linkage to the underlying cytoskeleton (Mostowy et al., 2011). However, it is not clear if septins affect MET degradation. Only some of the molecular events underlying the protective effects of septins against protein ubiquitylation and degradation are understood (Table 1), and further studies are required to determine the detailed mechanisms of septin dependent protein stabilization.

Understanding the mechanism by which septins contribute to intracellular stability of LCA would provide valuable insights for treating BoNT intoxication. On the other hand, the remarkable stability of LCA is fundamental in the success of BoNT/A for long-term treatment of several disorders as well as cosmetic therapies (Bhidayasiri and Truong, 2005; Chancellor et al., 2013; Esquenazi et al., 2013; Hallett et al., 2013; Naumann et al., 2013; Jost et al., 2015; Choi et al., 2016), and a super-stable LCA would be useful therapeutically.

A better understanding of septin contribution to the abnormal persistence of EGFR and ErbB2 in cancer cells will provide a potential treatment target for aggressive malignancies. Receptor-targeted therapies, such as monoclonal antibodies (e.g., trastuzumab), and tyrosine kinase inhibitors (e.g., gefitinib), are effective against several types of malignancies, but tumors may develop resistance to these agents due to compensatory mechanisms (Hynes and Lane, 2005; Takeuchi and Ito, 2011; Arteaga and Engelman, 2014), emphasizing the evolving need to develop new synergistic treatment strategies.

Knowledge of the pathways of septin contribution in oxygen-independent stabilization of HIF-1α would provide a better understanding of the reasons for HIF-1α over-expression in various cancers even in aerobic conditions, which correlates with poor prognosis, making HIF-1α an important target for cancer therapy. A better understanding of septin-mediated stabilization of HIF-1α opens the way to new therapeutic approaches to target “normoxic” tumor cells (Semenza, 2012; Burroughs et al., 2013; Warfel and El-Deiry, 2014).

## Author contributions

OV and DB wrote, revised, and approved the final version of the manuscript.

## Funding

Supported by National Heart, Lung, and Blood Institute (NHLBI) grant R01HL113350 (OV).

### Conflict of interest statement

The authors declare that the research was conducted in the absence of any commercial or financial relationships that could be construed as a potential conflict of interest.
